# Arginine Methyltransferase PRMT7 Deregulates Expression of RUNX1 Target Genes in T-Cell Acute Lymphoblastic Leukemia

**DOI:** 10.3390/cancers14092169

**Published:** 2022-04-26

**Authors:** Laura Oksa, Artturi Mäkinen, Atte Nikkilä, Noora Hyvärinen, Saara Laukkanen, Anne Rokka, Pekka Haapaniemi, Masafumi Seki, Junko Takita, Otto Kauko, Merja Heinäniemi, Olli Lohi

**Affiliations:** 1 Tampere Center for Child, Adolescent, and Maternal Health Research, Faculty of Medicine and Health Technology, Tampere University, FI-33520 Tampere, Finland; artturi.makinen@tuni.fi (A.M.); atte.nikkila@tuni.fi (A.N.); noora.hyvarinen@tuni.fi (N.H.); saara.laukkanen@tuni.fi (S.L.); olli.lohi@tuni.fi (O.L.); 2 Fimlab Laboratories, Department of Pathology, Tampere University Hospital, FI-33520 Tampere, Finland; 3 Turku Bioscience Center, University of Turku and Åbo Akademi University, FI-20014 Turku, Finland; anne.rokka@bioscience.fi (A.R.); pekka.haapaniemi@bioscience.fi (P.H.); otkauko@utu.fi (O.K.); 4 Department of Cell and Molecular Biology, Karolinska Institutet, SE-17165 Solna, Sweden; masafumi.seki@ki.se; 5 Graduate School of Medicine, Kyoto University, Kyoto JP-606-8501, Japan; jtakita@kuhp.kyoto-u.ac.jp; 6 The Institute of Biomedicine, University of Eastern Finland, FI-70211 Kuopio, Finland; merja.heinaniemi@uef.fi; 7 Tays Cancer Center, Tampere University Hospital, FI-33520 Tampere, Finland

**Keywords:** Leukemia, T-ALL, arginine methylation, PRMT7, RUNX1

## Abstract

**Simple Summary:**

Approximately 15–25% of acute lymphoblastic leukemias (ALL) originate from T-lineage cells. The prognosis of T-ALL is less favorable than in B-ALL and patients experience more often relapse, which is associated with dismal survival. No established prognostic biomarkers exist for T-ALL. Here, we identified the high expression of *PRMT7* in T-ALL cells. Genetic deletion of *PRMT7* decreased the colony formation of T-ALL cells and altered arginine monomethylation patterns in protein complexes associated with RNA and DNA processing. Moreover, several proteins with an established role in the pathogenesis of T-ALL had disrupted arginine monomethylation patterns. Among them was RUNX1, whose target gene expression was consequently deregulated.

**Abstract:**

T-cell acute lymphoblastic leukemia (T-ALL) is an aggressive hematological malignancy with no well-established prognostic biomarkers. We examined the expression of protein arginine methyltransferases across hematological malignancies and discovered high levels of *PRMT7* mRNA in T-ALL, particularly in the mature subtypes of T-ALL. The genetic deletion of *PRMT7* by CRISPR-Cas9 reduced the colony formation of T-ALL cells and changed arginine monomethylation patterns in protein complexes associated with the RNA and DNA processing and the T-ALL pathogenesis. Among them was RUNX1, whose target gene expression was consequently deregulated. These results suggest that PRMT7 plays an active role in the pathogenesis of T-ALL.

## 1. Introduction

T-cell acute lymphoblastic leukemia (T-ALL) is an aggressive hematological malignancy that accounts for approximately 15–25% of pediatric and adult ALL [1]. In contrast to B-cell acute lymphoblastic leukemia (B-ALL), the prognosis of T-ALL is less favorable despite significant improvements during the last decade [2]. Five-year event-free survival (EFS) and overall survival (OS) were 74% and 75% in the NOPHO ALL2008 study [3], to 76.3% and 81.2% in the AIEOP-BFM-ALL2000 study [4], 83.8% and 89.5% in the AALL0434 study [5], and 84.6% and 90.9% in the UKALL2003 study [6]. The prognosis differs among age groups, as the five-year EFS was 80%, 74%, and 64% for age groups 1–9, 10–17, and 18–45 years in the NOPHO ALL2008 study, respectively, and showed 15–20% improvement compared to previous protocols in the study group [3]. T-ALL patients more often experience failure of induction therapy or suffer from an early relapse, which are associated with a dismal prognosis [2,3]. In the NOPHO ALL2008 study, 14% of T-ALL patients relapsed. Overall, only three out of the 39 relapsed patients were alive at the time of the last follow-up [3]. Hence, there is an evident clinical need for more efficient therapies, especially for relapsed and refractory T-ALL.

Protein Arginine Methyltransferase 7 (PRMT7) belongs to the PRMT family, which consists of nine arginine methyltransferases [7,8,9]. In eukaryotic cells, they catalyze reactions where a methyl group from the S-adenosyl-L-methionine is transferred to an arginine residue on protein substrates. Methylation of arginine residue increases the bulkiness of the side chain of the protein, decreasing the hydrogen bonding potential and altering the binding of methylarginine to protein modules that read the arginine methylation marks, such as the PHD finger, Tudor domain, and SH3 domain [10,11,12,13,14].

PRMT7 is classified as a type III arginine methyltransferase because it generates only ω-monomethyl arginine (MMA) residues to its substrates, typically at basic RXR sequences in peptides and histones. In contrast to other PRMT enzymes, PRMT7 has two AdoMet-binding sites [7] and participates in many cellular processes, including transcriptional regulation, DNA damage repair, RNA splicing, cell differentiation and proliferation, and ability to metastasize, all of which are altered in cancer [8,9,15,16]. In breast cancer, renal cell carcinoma, and non-small-cell lung cancer, the high expression of PRMT7 has been associated with metastasis or decreased survival [15,16,17,18].

PRMT7 is an attractive target protein, as there are potential inhibitors available. A cell-active chemical probe inhibited the activity of PRMT7, leading to the silencing of HSP70 family proteins [19]. Another compound, a dual PRMT5-PRMT7 inhibitor, DS-437, decreased the cell proliferation and migration of human breast cell cancer cells (MDA-MB-231) by inhibiting PRMT5 and PRMT7 activity [20].

The role of PRMT enzymes in hematological malignancies is poorly characterized. Therefore, we examined the expression of the PRMT enzymes across hematological malignancies, particularly leukemias, and focused on the role of PRMT7 in T-ALL pathogenesis and prognosis.

## 2. Materials and Methods

### 2.1. Gene Expression Analysis

The HEMAP interactive online resource (http://hemap.uta.fi/, accessed on 1 March 2019) includes gene expression data from microarray studies of various hematological and lymphoid malignancies and healthy tissues performed on a uniform technical platform (Affymetrix HG U133 Plus 2.0) [21,22]. The clustering of different T-ALL subgroups was performed as described previously by Laukkanen et al. [23,24]. The Human Cell Atlas Developmental thymus data portal (https://developmentcellatlas.ncl.ac.uk/datasets/HCA_thymus/, accessed on 9 June 2020) was used to characterize *PRMT7*, *ILR7*, *MYC*, *MYB*, *GATA3*, *TAL1*, *NOTCH1*, and *RAG2* gene expression during human T-cell development [25].

### 2.2. Protein Expression and Immunohistochemistry

For protein extraction, cells were lysed with CellLytic M reagent (Sigma-Aldrich, Saint Louis, MO, USA) according to the manufacturer’s instructions. Protein samples were subjected to Mini-PROTEAN^®^ TGX Stain-Free™ Precast 10% or 12% gels (Bio-Rad) and transferred to 0.2 µM PVDF membranes using the Trans-Blot Turbo Transfer Pack and Trans-Blot Turbo Transfer System (Bio-Rad). Membranes were processed using standard procedures, with PRMT7 (1:1000 in 3% BSA, #14762, Cell Signaling Technology, Danvers, MA, USA), Histone H3 (1:10,000 in 3% BSA, #9715S, Cell Signaling Technology, Danvers, MA, USA), and Horseradish peroxidase conjugated anti-rabbit (1:5000 in 3% BSA, #7074S, Cell Signaling Technology, Danvers, MA, USA) antibodies. Cell Amersham ECL Reagent (GE Healthcare, Chicago, IL, USA) was used for the chemiluminescence reaction, and chemiluminescence was detected with ChemiDoc^TM^ XRS+ using Image Lab^TM^ Software (Bio-Rad, version 6.0.1, Hercules, CA, USA). PageRuler Plus prestained protein ladder (Thermo Fisher Scientific, Waltham, MA, USA) was used as a reference for protein size. Quantitation was performed using ImageJ software (version 1.59i) [26].

For the immunohistochemistry (IHC) of cell lines, cell pellets were collected from ~50 million cells via formalin fixation and paraffin embedding (FFPE). Tissue microarrays (TMA) of decalcified FFPE bone marrow trephine samples of pediatric ALL patients were used for IHC as previously described [27]. Briefly, the PRMT7 antibody (1:1000, #14762, Cell Signaling Technology, Danvers, MA, USA) and Ultraview Universal DAB kit were used for immunostaining. Then, the stained glass slides were scanned with the Hamamatsu Nanozoomer XR using a 40× magnification, and QuPath software (version 0.2.3) [28]) was applied for image analysis and the quantification of PRMT7 expression in leukemic blasts.

### 2.3. Cell Culture and Knockout Lines

CRISPR guide RNAs were designed with the Desktop genetics online tool (London, UK) and cloned into GFP fluorochrome marker containing vector px321-GFP (PRMT7-2: 5′-TGAACACTATGATTACCACCAGG-3′, PRMT7-3: 5′-ACCACCAGGAGATTGCAAGG-3′, PRMT7-5: 5′-AAGGCCTTGGTTCTCGACAT-3′). Jurkat cells, which stably express Cas9 enzyme (Jurkat-Cas9), were transfected with 2 µg of the plasmids (EV, PRMT7-2, PRMT7-3, and PRMT7-5) using 4D Nucleofector electroporation equipment (Lonza) and electroporation program CL-120 with the SE solution. After 24 h, GFP positive cells were single-cell sorted into 96-well plates in conditioned media that contained 20–40% of media harvested from cultured Jurkat and Jurkat-Cas9 cells and 60–80% RPMI 1640 media. *PRMT7* knockout cells for the Molt-4 line were obtained from Synthego (Synthego Corporation, Menlo Park, CA, USA). The bulk cells were single-cell sorted into 96-well plates in conditioned media containing 40% of media harvested from unmodified and modified Molt-4 cells and 60% of RPMI-1640 media. Single-cell clones were screened for mutations with T7 endonuclease (New England Biolabs, Ipswich, MA, USA) and confirmed by Sanger sequencing (DNA sequencing and genomics service, University of Helsinki, Helsinki, Finland). MutationTaster2 was used to study the functional effects of the mutations and to predict the disease-causing potential of DNA variants [29].

The Jurkat and Molt-4 cell lines and their derivatives were cultured in RPMI 1640 Medium (Gibco, Thermo Fisher Scientific, Waltham, MA, USA) with 2 mM L-glut, 100 U penicillin, and 100 µg/mL streptomycin with 10–20% FBS (Gibco). Cas9-expressing Jurkat cells were obtained from Jan Cool’s lab (VIB, Leuven, Belgium), whereas the unmodified and knockout cells from the Molt-4 line were purchased from Synthego (Synthego Corporation, Menlo Park, CA, USA). Mycoplasma testing was conducted on a regular basis for all cell lines, and STR genotyping was performed in Eurofins Genomics (Ebersberg, Germany) to authenticate the Jurkat Cas9 cell line.

### 2.4. Functional Studies

A Click-iT™ EdU Alexa Fluor™ 647 Flow Cytometry Assay (Invitrogen, Carlsbad, CA, USA) was used to study the cell cycle. Staining was performed according to the manufacturer’s instructions, adding 1 μL of EdU to 1 × 10^6^ cells in 1 mL and incubating at 37 °C for 1 h. Apoptosis was measured using the Annexin V Apoptosis Detection Kit-APC (eBioscience, Thermo Fisher Scientific) according to the manufacturer’s instructions. EdU and apoptosis assay results were recorded using BD FACS Canto II (BD Biosciences, San Jose, CA, USA).

For cell viability assay, 10,000 cells per cell line were seeded into a 96-well plate in 100 ul of growth media and incubated for the indicated times at 37 °C in 5% CO_2_. Cell viability was measured using a CellTiter-Glo Luminescent Cell Viability Assay (Promega, Madison, WI, USA) at the time points 0 h, 24 h, 48 h, and 72 h.

For colony formation analysis, MethoCult media was prepared by adding 600 µL of RPMI-1640 medium into 2.4 mL of methylcellulose-based MethoCult medium (H4230, Stemcell Technology). Cells were harvested and added into the MethoCult medium at a concentration of 10,000 cells/mL. Thereafter, 1.1 mL of MethoCult mixture containing cells were plated into 6-well plates with 1000 cells per well. Cells were incubated at 37 °C in a 5% CO_2_ incubator for 14 days. Colonies were imaged at day 10 or 14 with a Nikon AZ100 Fluorescence Macroscope (Nikon, Minato, Tokyo, Japan).

### 2.5. Mass Spectrometry-Based Arginine Monomethylation Analysis

For protein extraction, 1 × 10^8^ cells were collected from J-EV, J-KO3, and J-KO5 cells (*n* = 6). Proteins were extracted using a Urea lysis buffer (200 mM HEPES pH 8.0 (Sigma-Aldrich), 9 M urea (Thermo Fisher Scientific), 2.5 mM sodium orthovanadate (Sigma-Aldrich), 1 mM sodium pyrophosphate (Sigma-Aldrich), 1 mM β-glycerophosphate (Cayman Chemical, Ann Arbor, MI, USA)), and lysates were sonicated (1 min, pulse 1 sec on/off with 40% amplitude, three times) using a Vibracell 500-Watt ultrasonic processor (VC 505) sonicator (3 mm microtip) (Sonics & Materials Inc., Newtown, CT, USA) to break apart the DNA. Samples were processed according to PTMScan^®^ Mono-Methyl Arginine Motif [mme-RG] kit (Cell Signaling Technology) protocol. Shortly, proteins were digested by proteases, and the resulting peptides were purified by reversed-phase extraction. Peptides were then subjected to immunoaffinity purification. Unbound peptides were removed through washing, and the captured MMA-containing peptides were eluted with dilute acid. The enriched peptides were analyzed with a Q Exactive HF mass spectrometer (Thermo Fisher Scientific). Label free quantification was carried out in the peptide isoform level. Abundance values were calculated based on intensities of peptide precursor ions.

### 2.6. LC-MS/MS Analysis

The LC-ESI-MS/MS analyses were performed on a nanoflow HPLC system (Easy-nLC1200, Thermo Fisher Scientific) coupled to the Q Exactive HF mass spectrometer (Thermo Fisher Scientific, Bremen, Germany) equipped with a nano-electrospray ionization source. Peptides were first loaded on a trapping column and subsequently separated inline on a 15 cm C18 column (75 μm × 15 cm, ReproSil-Pur 3 μm 120 Å C18-AQ, Dr. Maisch HPLC GmbH, Ammerbuch-Entringen, Germany). The mobile phase consisted of water with 0.1% formic acid (solvent A) and acetonitrile/water (80:20 (*v*/*v*)) with 0.1% formic acid (solvent B). Peptides were eluted with a 120 min gradient: from 2% to 39% of solvent B in 105 min, from 39% to 100% solvent B in 10 min, followed by wash for 5 min at 100% of solvent B. MS data were acquired automatically by using Thermo Xcalibur 4.1. software (Thermo Fisher Scientific). An information dependent acquisition method consisted of an Orbitrap MS survey scan of mass range m/z 300–1750 followed by HCD fragmentation for the most intense peptide ions.

Database searches were performed using Proteome Discoverer 2.4 software (Thermo Fisher Scientific) connected to in-house Mascot 2.7.0 search engine software (version 2.7.9, Matrix Science, Boston, MA, USA) against the UniProt Swiss-Prot (version 2021_02) Homo sapiens protein database [30]. Search criteria were the following: trypsin as an enzyme; cysteine carbamidomethylation as fixed modification; arginine methylation, lysine methylation, methione oxidation, and N-terminal acetylation as variable modifications; peptide mass tolerance 10 ppm; and MS/MS ion tolerance 0.02 Da. Label free quantification, validation, and filtering of the results were performed using Proteome Discoverer 2.4 software (version 2.4, Thermo Fisher Scientific, Waltham, MA, USA).

### 2.7. Survival Analysis

The gene expression of *PRMT7* was explored in the context of patient survival from pediatric T-ALL patients in two different cohorts (TARGET, *n* = 264; Japanese leukemia group, *n* = 119) [31,32,33]. The Kaplan–Meier method was used to estimate survival and the group differences were tested by using the log-rank method. The events included induction failure, disease progression, relapse, secondary malignant disease, and death by any cause. The Cox proportional hazards model was used to examine the association of individual risk factors with patient survival. The proportionality assumption was tested with Schoenfeld residuals. The R-packages *survminer* and *survival* were used for data visualization and ordered expression graphs were plotted. The R (v. 3.6.2) software environment (R Foundation for Statistical Computing, Vienna, Austria) was used for statistical analyses.

### 2.8. RNA Sequencing

Library preparation and RNA sequencing of cell lines from unmodified Jurkat and Molt-4 cells and *PRMT7* knockout lines were performed by Novogene (Cambridge, UK). The quality of the raw sequencing reads was ensured with FastQC (v0.11.8). The reads were mapped to the human reference genome version hg19 using STAR aligner software (2.5.3a modified). Reads aligning to more than two locations were discarded. The alignment file was turned into tag directories, and read counts were calculated using the HOMER toolkit (v.4.11). Differential gene expression was analyzed using the quasi-likelihood F-test from edgeR R-package. Volcano plots were drawn using the R-package *ggplot2* Volcano plot function, combining the three biological replicates and using log2 fold changes values and FDR values [34].

### 2.9. Enrichment Analysis

Gene lists with up- and downregulated genes were analyzed using the online web server Enrichr (release March 2021) [35,36,37]. The analysis was performed based on gene sets from transcription factor (TF) perturbations followed by expression. In order to test whether the RUNX1 target gene expression was affected by the *PRMT7* KO, we specifically selected the significant RUNX1 related terms (GSE40155, GSE29639, GSE47375, GSE46970, GSE34292) (*p*-value < 0.05).

### 2.10. Statistical Analyses

The R (v. 4.0.2) software environment (R Foundation for Statistical Computing, Vienna, Austria) and Microsoft Excel (Redmond, WA, USA) were used in statistical analyses. The Wilcoxon test was used to compare the statistical significance of differences in gene expression of different leukemia types. All in vitro experiments were performed in three or more independent biological replicates, and the students’ *t*-test was used to analyze the statistical significance of differences. All statistical tests were two-tailed, and a *p*-value < 0.05 was considered statistically significant. In mass spectrometry-based arginine monomethylation analysis, statistical testing was performed with a two-tailed Student’s *t*-test, and *p*-values were adjusted following the Benjamini–Hochberg procedure [38] to account for multiple testing. Human KEGG pathway data were downloaded on 20 January 2022 from the Kyoto Encyclopedia of Genes and Genomes (KEGG) REST server using R package KEGGREST [39]. Human protein complexes were defined based on the CORUM database 3.0 release [40]. Unsupervised clustering was performed using Pearson’s correlation as distance metric and Ward’s linkage method.

## 3. Results

### 3.1. PRMT7 Is Strongly Expressed in Mature T-ALL

**Figure 1:**
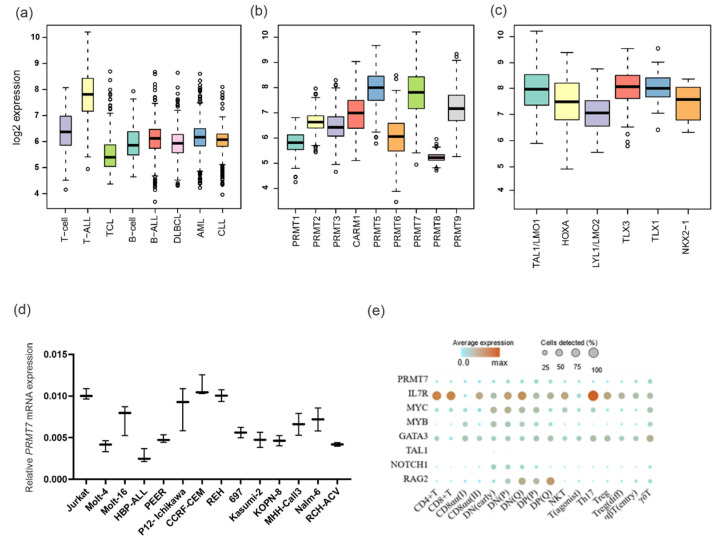
*PRMT7* expression in hematological malignancies and healthy T-cells. (**a**) Expression of *PRMT7* in leukemias (*n* = 4430), lymphomas (*n* = 1306), and healthy cells (*n* = 428) in Hemap dataset (*p*-values presented in Table S1); (**b**) summary of expression of all *PRMT* family genes in T-ALL; (**c**) expression of *PRMT7* in subgroups of T-ALL (*HOXA* vs. *TAL1/LMO1* *p* = 0.003, *HOXA* vs. *LYL1/LMO2 p* = 0.026, *HOXA* vs. *TLX3* *p* = 0.010, *HOXA* vs. *TLX1 p* = 0.006, *HOXA* vs. *NKX2-1 p* = 0.845, *LYL1/LMO2* vs. *HOXA p* = 0.025, *LYL1/LMO2* vs. *TAL1/LMO1 p* = 1.098 × 10^−9^, *LYL1/LMO2* vs. *TLX3 p* = 2.368 × 10^−7^, *LYL1/LMO2* vs. *TLX1 p* = 9.859 × 10^−8^, and *LYL1/LMO2* vs. *NKX2-1 p* = 0.198, *TAL1/LMO2 n* = 171, *HOXA n* = 56, *LYL1/LMO2 n* = 54, *TLX3 n* = 35 and *NKX2-1 n* = 11); (**d**) expression of *PRMT7* mRNA in T- and B-ALL cell lines as measured by qRT-PCR (*n* = 3).; and (**e**) expression of *PRMT7* and the selected essential T-cell developmental genes during the T-cell differentiation and maturation process at single-cell resolution. Color indicates the mean expression and size the proportion of cells expressing the gene relative to the absolute number of cells detected in the dataset.

We recently published global gene expression data of various hematologic malignancies and healthy tissues comprised of 9544 expression profiles, including 4430 leukemias (385 T-ALL, 1304 pre-B-ALL, 1713 acute myeloid leukemia (AML), and 801 chronic lymphocytic leukemia (CLL), 1306 lymphomas (208 T-cell lymphoma (TCL) and 743 diffuse large B-cell lymphoma (DLBCL), and 428 healthy samples (247 T-lymphocytes and 75 B-lymphocytes) (Hemap resource (http://hemap.uta.fi/hemap/index.html, accessed on 1 March 2019), [21,22]). Using this data set, we evaluated the expression of the *PRMT* family of genes in various leukemias, lymphomas, and healthy B- and T-lymphocytes. Overall, genes of the *PRMT* family exhibited moderate and relatively even expression across hematological malignancies, with few exceptions. *PRMT3* and *PRMT5* were markedly decreased in CLL, while *PRMT5* and *PRMT7* were increased in T-ALL (Figure 1a,b and Figure S1a). The expression of *PRMT7* was approximately 2.6-fold higher in T-ALL compared to healthy T-lymphocytes (*p*-value < 0.01), whereas *PRMT5* was 1.5-fold higher in T-ALL compared to T-lymphocytes (Figure 1a and Figure S1 and Table S1).

From here onwards, we focused our attention on *PRMT7* in T-ALL due to its strong expression in T-ALL in relation to healthy T cells. Among the genetic subgroups of T-ALL, *PRMT7* expression levels were significantly higher in the mature subtypes of T-ALL than in the more immature *LYL1/LMO2* and *HOXA*-associated T-ALL (Figure 1c). Out of the tested leukemia cell lines, T-ALL-derived cells had the highest level of *PRMT7* mRNA by RT-qPCR (Figure 1d).

At the protein level, PRMT7 expression was examined in 84 bone marrow biopsies that included T-ALL (*n* = 6) and B-ALL (*n* = 78). Two out of the six T-ALL cases showed strong staining, with 49% and 57% of the blasts positive, while two cases showed weak staining and two remained negative (Figure S1b). Weak to moderate expression of the PRMT7 protein was noted across subtypes of B-ALL (Figure S1b).

To determine whether the expression of *PRMT7* was present during the development and maturation of T-cells, we examined healthy T-lineage cells at the single-cell (sc) level. Here, we utilized a publicly available scRNA-seq data set that includes cells from the early double negative phase to the late CD4 or CD8 single positive phases, and retains mature T-regulatory, CD8αα+, and γδ T-cells [25]. Only weak expression of *PRMT7* was observed during the later stages of differentiation or maturation of T-cells (Figure 1e), suggesting that the strong expression of *PRMT7* in T-ALL is aberrant and associated with the leukemogenic process.

### 3.2. Association of PRMT7 Expression with Survival

**Figure 2:**
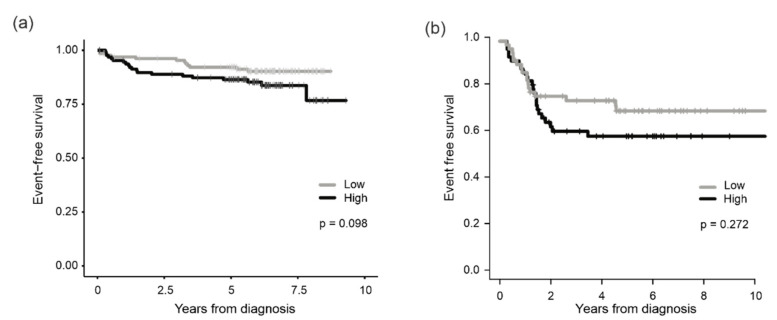
Association of *PRMT7* expression with patient outcome. EFS of patients in (**a**) the TARGET and (**b**) the Japanese patient cohorts. Patients were classified into two groups by using the median expression value of *PRMT7* as a cut-off.

We next examined whether the level of *PRMT7* mRNA was associated with patient survival using two independent patient cohorts. In the TARGET data set, which consisted of 264 T-ALL patients, *PRMT7* expression was categorized into two groups by using the median value as a cut-off. Cases with above-median expression level (*PRMT7*^high^) had a 5-y EFS of 86% (95% CI, 81–93%), while cases with below-median level (*PRMT7*^low^) had an EFS of 92% (95% CI, 88–97%) (*p* = 0.098) (Figure 2a, Table S2).

In another patient cohort that included 119 pediatric T-ALL patients, a similar prognostic trend was observed: patients with *PRMT7*^high^ had worse EFS (Figure 2b) and increased hazard for an event (HR = 1.41, 95% CI 0.76–2.6) (*p* = 0.272). Neither of the survival differences reached statistical significance.

The Cox proportional hazard model was used to assess the *PRMT7* mRNA levels in conjunction with the known T-ALL risk factors (age, white blood cell count, and minimal residual disease). In the univariate model, *PRMT7*^high^ showed a trend to increased hazard for an event (HR = 1.81, 95% CI, 0.89–3.71, *p* = 0.1), but it did not reach statistical significance. A similar trend was observed in the multivariate model (HR = 1.77, 95% CI, 0.85–3.68, *p* = 0.13) (Table S2).

### 3.3. Reduced Colony Formation and Cell Viability after Genetic Deletion of PRMT7

**Figure 3:**
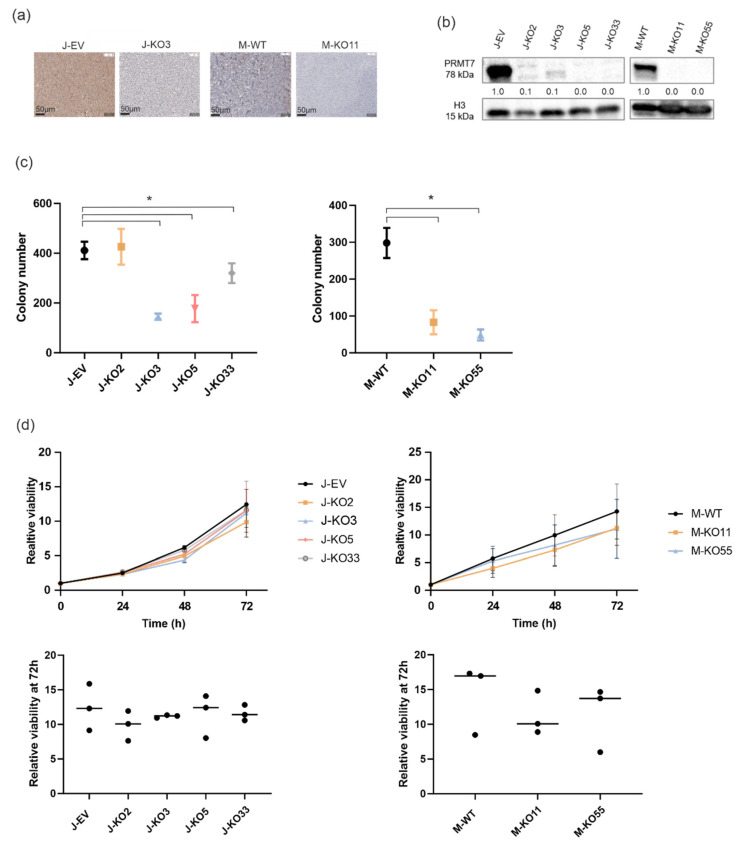
Functional consequences of genetic deletion of *PRMT7* in T-ALL cells. (**a**) Immunohistochemical staining (scale bar 50 µm) showing PRMT7 expression in J-EV, J-KO3, M-WT and M-KO11 cell lines. Brown color indicates positivity to the PRMT7 protein and blue marks PRMT7 negative cells. (**b**) Western blotting in unmodified (J-EV and M-WT) and knockout cell lines (marked with KO) by using the PRMT7 antibody. Quantitation in Western blot is relative to J-EV or M-EV control cell lines. J stands for Jurkat and M for Molt4 cell line, EV for empty vector and WT for wild type. The uncropped Western blots have been shown in Figure S6. Effect of the *PRMT7* knockout on (**c**) colony formation (*n* = 4) and (**d**) cell viability (*n* = 3). Cell viability results are presented as line plots for all the examined time points and as scatter plots for the 72 h time point. Asterisks denote samples with significant *p*-values (<0.05) when compared to the unmodified (J-EV and M-WT) cells.

Aberrant expression of *PRMT7* in T-ALL might indicate a role in leukemogenic process. Hence, we generated several knockout (KO) cells using CRISPR-Cas9 in two T-ALL cell lines, Jurkat and Molt-4. Guide RNAs targeting the first exon of the *PRMT7* gene led to indel mutations that introduced early stop codons in the coding sequence of *PRMT7* (Figure S2). Abrogation of the PRMT7 protein expression was confirmed by immunohistochemistry and Western blotting (Figure 3a,b and Figure S3a).

A colony formation assay was performed to study the clonogenic capacity of Jurkat empty vector (J-EV) transduced, Molt-4 wild type (M-WT), and *PRMT7* KO cell lines. Except for one line (J-KO2), all *PRMT7* KO cells showed a significantly decreased number of colonies (*p*-values < 0.05) when compared to the control lines (Figure 3c and Figure S3b). In the cell viability, apoptosis, and cell cycle assays, variation in results was evident across the Jurkat and Molt-4 derived KO lines, with no consistent changes in either direction (Figure 3d and Figure S3b–d). The variability of the results may have been caused by residual protein expression, as shown in a report by Smith et al. (2019) [41].

### 3.4. Arginine Monomethylation Is Disrupted after PRMT7 Knockout in T-ALL

**Figure 4:**
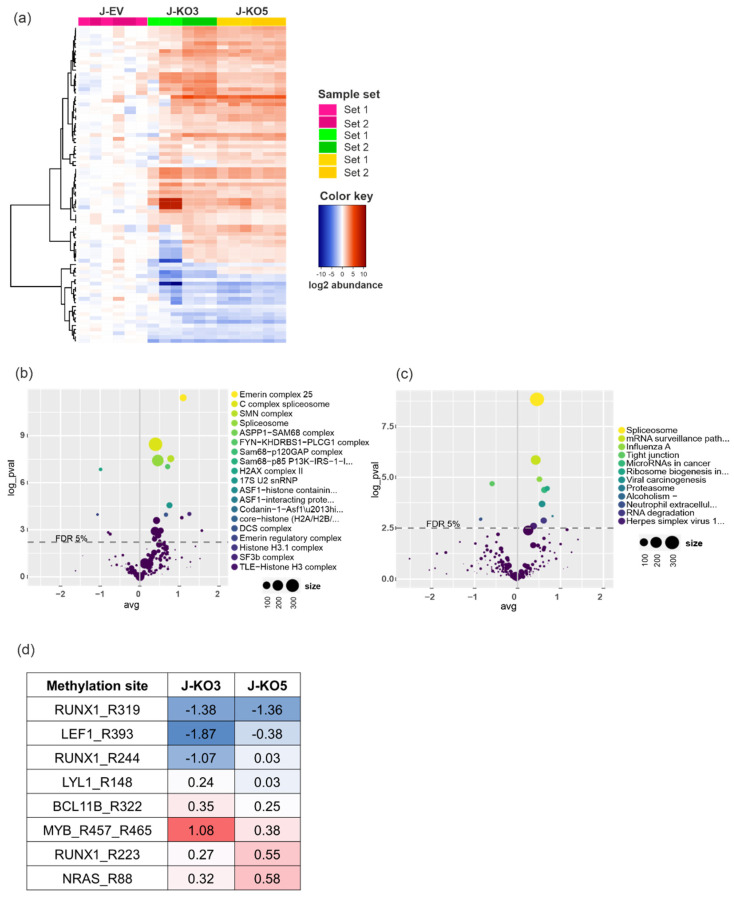
Effects of *PRMT7* knockout on arginine monomethylation in T-ALL cells. (**a**) A heatmap of top differentially regulated peptides with altered arginine monomethylation level in *PRMT7* knockout cells (J-KO3 and J-KO5) compared to unmodified cells (J-EV) (Benjamini-Hochberg adjusted *p*-value ≤ 0.01). Color key indicates the average arginine monomethylation in log2 abundance. Duplicate image with row names is presented in Figure S4c. Impact of *PRMT7* knockout to arginine monomethylation by (**b**) CORUM protein complexes (Benjamini-Hochberg adjusted log2 *p*-value < 0.05) and (**c**) KEGG pathways (Benjamini-Hochberg adjusted log2 *p*-value < 0.05), with both cell lines (J-KO3 and J-KO5) combined for the analysis. (**d**) Tabulation of arginine monomethylation changes in peptides belonging to proteins associated with T-ALL pathogenesis in *PRMT7* knockout cell lines (J-KO3 and J-KO5). Red color indicates increased and blue decreased level of arginine monomethylation compared to unmodified cells.

PRMT7 methylates arginine residues in histone and non-histone proteins in a monomethyl manner [7]. Hence, we performed arginine monomethylation mapping in control Jurkat cells (J-EV) and Jurkat-derived *PRMT7* KO cells (J-KO3 and J-KO5). Monomethylation analysis was performed in two sets and included three biological replicates for each cell line. After the normalization of peptides to the median abundance of control, sample replicates clustered well in both sample sets and displayed high correlation, indicating the similarity of the knockout cell lines (Figure 4a and Figure S4, Table S3). Hence, for the downstream analysis, the J-KO3 and J-KO5 data sets were combined.

We first examined global alterations in arginine monomethylation patterns in protein complexes by utilizing the Comprehensive Resource of Mammalian protein complexes (CORUM) database [40]. Average peptide methylation was statistically significantly changed in 37 protein complexes out of 243 (Benjamini–Hochberg adjusted *p*-value < 0.05) (Figure 4b, Table S4). Most of the differentially regulated peptides belonged to protein complexes affecting RNA and DNA processing. In total, 19 protein complexes had functional effects on RNA binding, splicing, processing, biosynthesis, or transcriptional regulation (e.g., Emerin complex 25 and C complex spliceosome), with average methylation of 18 complexes increased compared to the control cells. Complexes having a role in DNA processing, such as DNA binding, replication, repair, and synthesis, or chromatin remodeling (e.g., AFF1-histone containing and ASF1-interacting protein complex) had mostly decreased average arginine monomethylation levels. We also found methylation changes in two complexes, which play an essential role in tumor development, namely H2AX complex II and PIDDsome [42,43], both having decreased average arginine monomethylation levels in *PRMT7* KO cells.

To complement the methylation analysis, we applied the Kyoto Encyclopedia of Genes and Genomes (KEGG) database [39]. Average peptide methylation level was significantly altered in 12 pathways (Benjamini–Hochberg adjusted *p*-value < 0.05), with an increase in eight and decrease in four pathways (Figure 4c, Table S5). Four of the pathways with increased methylation levels were associated with RNA processing, including spliceosome, mRNA surveillance, ribosome biogenesis, and RNA degradation pathways.

We next evaluated changes in arginine monomethylation patterns in proteins that have a well-established role in the pathogenesis of T-ALL [44]. Of particular interest were recent reports showing that changes in RUNX1 arginine methylation alter its transcriptional activity [45,46]. Indeed, *PRMT7* KO cells displayed changes in several peptides belonging to RUNX1, including a decrease in monomethylation at residue R319, a decrease or unaltered status at residue R224, and an increase at residue R223. Moreover, changes were observed in arginine monomethylation levels in peptides belonging to LEF1, LYL1, BCL11B, MYB, and NRAS proteins, all critical players in T-ALL (Figure 4d).

### 3.5. PRMT7 Deletion Deregulates Expression of RUNX1 Target Genes

**Figure 5:**
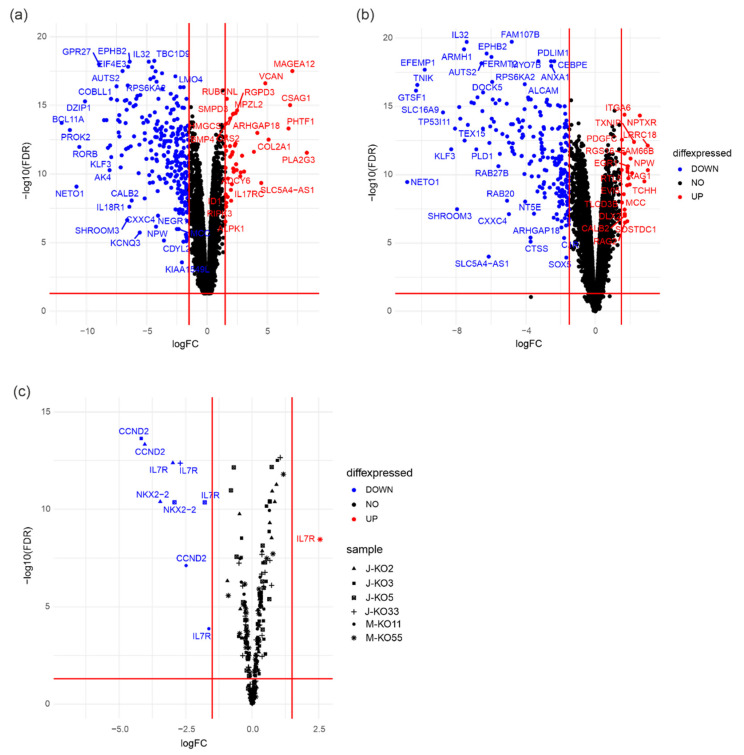
Effects of *PRMT7* deletion on gene expression in T-ALL cells. Volcano plots displaying the differentially expressed genes (log2FC ≥ ±1.5, FDR < 0.05) in (**a**) J-KO3 and (**b**) J-KO5 cell lines. (**c**) A volcano plot displaying gene expression changes (log2FC ≥ ±1.5, FDR < 0.05) in genes known to be associated with T-ALL pathogenesis and altered in all six *PRMT7* knockout cell lines labeled with different symbols. Red color indicates genes with increased and blue with decreased expression compared to unmodified cells. Three biological replicates were combined for the analysis for each cell line. J stands for Jurkat and M for Molt4 cell line, EV for empty vector and WT for wild type.

As monomethylation of arginine residues can lead to altered gene expression, we performed RNA sequencing of the knockout and control cells. In Jurkat-derived *PRMT7* KO lines, downregulation of 23 genes was noticed in all four knockout lines, and in 65 genes in at least three cell lines (log2FC ≥ ±1.5, FDR < 0.05) (Figure 5a and Figure S5a,b). Among the downregulated genes were *BCL11A* and *DNTT*, both well-known regulators of T-cell development. BCL11A acts as an oncogene in hematological malignancies, while DNTT plays role in early T-cell differentiation and V(D)J recombination [47,48,49,50,51]. Genes involved in propagating tumorigenesis and cell growth, namely *MDK* and *EPHA3* [52,53], or drug resistance and relapse in T-ALL, *ST8SIA6* and *NR3C2* [54,55,56], were silenced in the *PRMT7* KO lines. In Molt-4-derived *PRMT7* KO lines, downregulation of 24 genes was seen in both cell lines (log2FC ≥ ±1.5, FDR < 0.05) (Figure 5b and Figure S5c,d). When data on the Jurkat- and Molt-4-derived knockout lines were combined, four genes, namely *ARHGEF26* (also known as *SGEF*), *AUTS2*, *EPHA3*, and *MLC1*, were consistently silenced across all lines. Only genes in two Jurkat-derived PRMT7 KO cell lines were upregulated, i.e., MCC and NEDD9. Upregulation of MCC is associated malignant cell transformation for example in B-cells, and NEDD9 is associated to oncogenic signaling and tumor aggressiveness [57,58,59].

When focusing on the expression of important T-ALL associated genes, we noticed the silencing of *IL7R* gene, which harbors gain-of-function mutations in around 10% of T-ALL leading to the constitutive activation of downstream signaling [60], in five out of six *PRMT7* KO lines (log2FC ≥ ±1.5, FDR < 0.05) (Figure 5c). Moreover, *PRMT7* KO led to the downregulation of cyclin D2 (*CCDN2*), which complexes with CDK4 and CDK6 to progress the cell cycle trough G1/S transition, and *NKX2-2*, a gene encoding a transcriptional activator protein (Figure 5c) [61,62,63].

Due to changes in the monomethylation of three arginine residues in the RUNX1 protein, we specifically asked if the expression of RUNX1 target genes was affected by the *PRMT7* deletion. Indeed, Enrichr analysis [34,35,64] revealed that in the *PRMT7* KO lines five RUNX1 target gene sets were statistically significantly changed (log2FC ≥ ±1.5, *p*-value < 0.05) (Table S6).

## 4. Discussion

T-ALL is an aggressive leukemia that accounts for approximately 10–15% of pediatric and 25% of adult ALL cases and lacks well-established prognostic biomarkers. We discovered the high expression of *PRMT7* in T-ALL compared to healthy T-cells. Jurkat and Molt-4 T-ALL cells lacking the *PRMT7* gene had diminished colony formation capability. Moreover, alterations in the monomethylation of arginine residues were observed in proteins with a well-known role in T-ALL, including RUNX1, whose target gene expression was consequently deregulated.

The proteins of the PRMT family methylate arginine residues and thereby affect many cellular processes, such as gene transcription, RNA splicing, and DNA damage response [11,14,65,66,67,68,69]. The methylation of arginine in histone proteins plays an important role in fine-tuning gene expression [70] and it is no surprise that it plays an important role in cancer as well. The high expression of PRMT1 has been shown to block cell differentiation and propagate leukemia, whereas PRMT4 (CRAM1) and PRMT5 affected RNA splicing in hematological malignancies [14,71]. Aberrant expression of PRMT7 has been linked with altered RNA splicing, proliferation, and colony formation in breast, colorectal, and prostate cancers [14,16]. To date, no mutations have been identified in the PRMT genes that could explain their aberrant expression in cancers [71,72].

Previously, no comprehensive studies have been conducted on *PRMT7* in leukemia and lymphoma. We found that *PRMT7* was highly expressed in T-ALL, particularly in the more differentiated subtypes when compared to healthy T-cells. Weak expression was observed during the later stages of development of healthy T-cells, indicating that the strong expression in immature T-lymphoblasts is brought about by disease associated events. Two independent patient cohorts demonstrated a decreased survival trend in patients with high levels of *PRMT7* mRNA expression, although the difference did not reach statistical significance, possibly due to the low number of events in the cohorts. It is noteworthy that the TARGET cohort excluded patients with over 5% residual disease at the end of the induction period, thus leaving out the patients with the worst prognosis. Survival results align well with our cell modelling data, where deletion of the *PRMT7* gene decreased the oncogenic potential of the T-ALL cells as measured by colony formation assay.

As PRMT7 catalyzes the transfer of methyl groups into arginine residues, we performed global monomethyl mapping in *PRMT7* knockout cells. The most significantly affected protein complexes were involved in the RNA and DNA processing. Surprisingly, many of the complexes affecting RNA binding, splicing, biosynthesis, or transcription had increased average arginine methylation in *PRMT7* KO cells, suggesting that other enzymes with similar activity may have compensated for the loss, and perhaps in a slightly different manner. PRMT family enzymes do not exhibit strong sequence selectivity since for example PRMT5 can methylate the same proteins as PRMT7 [14]. Arginine methylation can prevent hydrogen bonding by sterically hindering the interactions between RNA and protein, thereby negatively affecting the affinity of a particular RNA-binding protein to its target. Alternatively, arginine methylation can positively regulate RNA–protein interactions, making the arginine more “hydrophobic”, and thereby facilitate stacking of the bases of the RNA [73,74]. On the other hand, a majority of the complexes associated with DNA or chromatin remodeling had decreased levels of arginine monomethylation in *PRMT7* KO cells. The involvement of arginine methylation in the DNA damage response and DNA repair may cause conformational changes in DNA and trigger genomic instability [73].

We also examined the arginine monomethylation in proteins that have a well-established role in the T-ALL pathogenesis [44] and noticed minor changes in peptides belonging to LEF1, LYL1, BCL11B, MYB, and NRAS proteins. However, the most noticeable finding was associated with the RUNX1 protein, in which three arginine residues showed altered monomethylation levels. Simultaneously, the expression of the RUNX1 target genes was significantly deregulated, suggesting that modifications by PRMT7 may play an important role in fine-tuning transcriptional activity of the RUNX1 protein. This may have significant relevance in T-ALL as RUNX1 targets are involved in many critical cellular processes, especially in hematopoietic cells, such as cell differentiation, ribosome biogenesis, cell cycle regulation, and p53 and transforming growth factor beta (TGF-β) signaling pathways [29,75,76]. RUNX1 is also critical for the differentiation of lymphocytes, and its aberrant expression has been linked with various hematological malignancies [31,77]. Previously, arginine monomethylation of RUNX1 by PRMT1 in hematopoietic stem cells was associated with resistance to apoptosis and increased cell survival [46,62,78]. In myeloid cells, methylation of RUNX1 protein at residue R233 by the PRMT4 protein led to the decreased translation of the myeloid target genes, manifested as the persistent maintenance of an undifferentiated phenotype of hematopoietic progenitor cells [45].

Our results suggest that a high level of *PRMT7* may augment the aggressiveness of T-ALL. To this end, we sought an answer by looking at RNA-sequencing data in the *PRMT7* knockout cell lines. Many genes involved in drug resistance, tumor progression, or T-ALL pathogenesis were silenced in the *PRMT7* knockout cells, suggesting that the high expression of *PRMT7* is relevant for their expression. Among the silenced genes was *BCL11A*, whose overexpression is observed in many hematological cancers and mediates its oncogenic role in leukemia via target genes *Bcl2*, *Bcl2-xL*, *Mdm2*, and *p53* [47,48]. We also discovered the downregulation of three genes (*NR3C2*, *ST8SIA6*, and *ARHGEF26*) in the *PRMT7* knockout cells involved in drug resistance and relapse. NR3C2 is a glucocorticosteroid receptor and associated with NR3C1 in glucocorticoid response [56]. ST8SIA6 is a member of sialytransferases (STs), and aberrant sialylation of the cell surface glycoprotein is linked with multidrug resistance in AML and CML [54,55]. Likewise, the reduction of *ARHGEF26* sensitizes cells to chemotherapeutic agents and suppresses the colony formation of glioma cells [79]. We also observed changes in the expression of *IL7R*, *CCDN2*, and *NKX2-2*, which all have an important role in the T-ALL pathogenesis by participating in the cell cycle, growth, and transformation [60,61,62,63].

## 5. Conclusions

The high expression of *PRMT7* was associated with increased oncogenicity in vitro and a trend towards an inferior outcome in T-ALL in two independent patient cohorts. Alterations of arginine monomethylation after the genetic deletion of *PRMT7* were accompanied by changes in the target gene expression of the RUNX1 protein. These results suggest that PRMT7 plays an active role in the pathogenesis of T-ALL and further mechanistic studies are warranted in future.

## Data Availability

The RNA-seq data generated and analyzed in the current study are available in the Gene Expression Omnibus (GEO) repository: GSE148658 (Jurkat EV control cells) GSE186238 (Jurkat Cas9 *PRMT7* KO2, Jurkat Cas9 *PRMT7* KO3, Jurkat Cas9 *PRMT7* KO5, Jurkat Cas9 *PRMT7* KO33, Molt-4 *PRMT7* KO11 and Molt-4 *PRMT7* KO55 cells). Raw MS data (Jurkat Cas9 EV control, Jurkat Cas9 *PRMT7* KO3 and Jurkat Cas9 *PRMT7* KO5 cells) is available at the jPOSTrepo (Japan ProteOme STandard Repository) with jPOSTrepo identification number JPST001540 and ProteomeXchange identification number PXD032819 [80]). Data sets reanalyzed in this study are available at: http://hemap.uta.fi/ (Hemap resource, accessed on 1 March 2019), https://portal.gdc.cancer.gov/projects (TARGET, accessed on 8 November 2019), https://developmentcellatlas.ncl.ac.uk/(Human Cell Atlas Developmental; thymus data portal accessed on 2 September 2019).

## Supplementary Materials

The following supporting information can be downloaded at: https://www.mdpi.com/article/10.3390/cancers14092169/s1, Figure S1: Boxplots presenting expression of the *PRMT* family of genes in leukemias, lymphomas, and healthy cells, and in T- and B-lineage cell lines, Figure S2: Schematic representation of the consequences of CRISPR-Cas9-mediated genetic mutations of *PRMT7* in the Jurkat and Molt-4 cell lines, Figure S3: Images of effects of *PRMT7* knockout on colony formation, and bar plots presenting effect on apoptosis, and cell cycle, Figure S4: Plots illustrating the correlation of arginine monomethylation findings in two PRMT7 knockout cell lines (J-KO3 and J-KO5) when using the CORUM and KEGG databases for the analysis, Figure S5: Volcano plots showing effects of *PRMT7* knockout on gene expression of T-ALL cells, Figure S6: The uncropped Western blots, Table S1: Results of statistical test from HEMAP analysis where PRMT family gene expressions in T-ALL compared to other cell types, p-values are calculated using Wilcoxon rank sum test, Table S2: Multivariate and univariate analyses of the event-free survival based on the expression level of PRMT7 in pediatric T-ALL from the TARGET data set, Table S3: Top differentially regulated peptides (FDR ≤ 0.01) normalized to the median abundances of control using Pearson’s correlation, Table S4: Average arginine monomethylation (abundance ratio) of CORUM protein complexes in PRMT7 KO cells (J-KO3 and J-KO5 cell lines combined for the analysis), Table S5: Average arginine methylation (abundance ratio) of KEGG pathways in PRMT7 KO cells (J-KO3 and J-KO5 cell lines combined for the analysis), Table S6: Up- and downregulated genes in PRMT7 KO cells analyzed using Enrichr. Significant terms (*p*-value < 0.05) summarized from TF perturbations followed by expression. Yellow color highlights the RUNX1 related gene sets.
