# Supplementary material for: Arginine Methyltransferase PRMT7 Deregulates Expression of RUNX1 Target Genes in T-Cell Acute Lymphoblastic Leukemia
Source: Cancers (Basel). 2022 Apr 26;14(9):2169. doi: 10.3390/cancers14092169 (PMC9101393; doi:10.3390/cancers14092169)
Supplement: Supplementary file 1 [file cancers-14-02169-s001.zip › cancers-1664799-supplementary figures.pdf]

cancers MDPI

Arginine Methyltransferase PRMT7 Deregulates Expression of RUNX1 Target Genes in T-Cell Acute Lymphoblastic Leukemia

Laura Oksa, Artturi Mäkinen, Atte Nikkilä, Noora Hyvärinen, Saara Laukkanen, Anne Rokka, Pekka Haapaniemi, Masafumi Seki, Junko Takita, Otto Kauko, Merja Heinäniemi and Olli Lohi

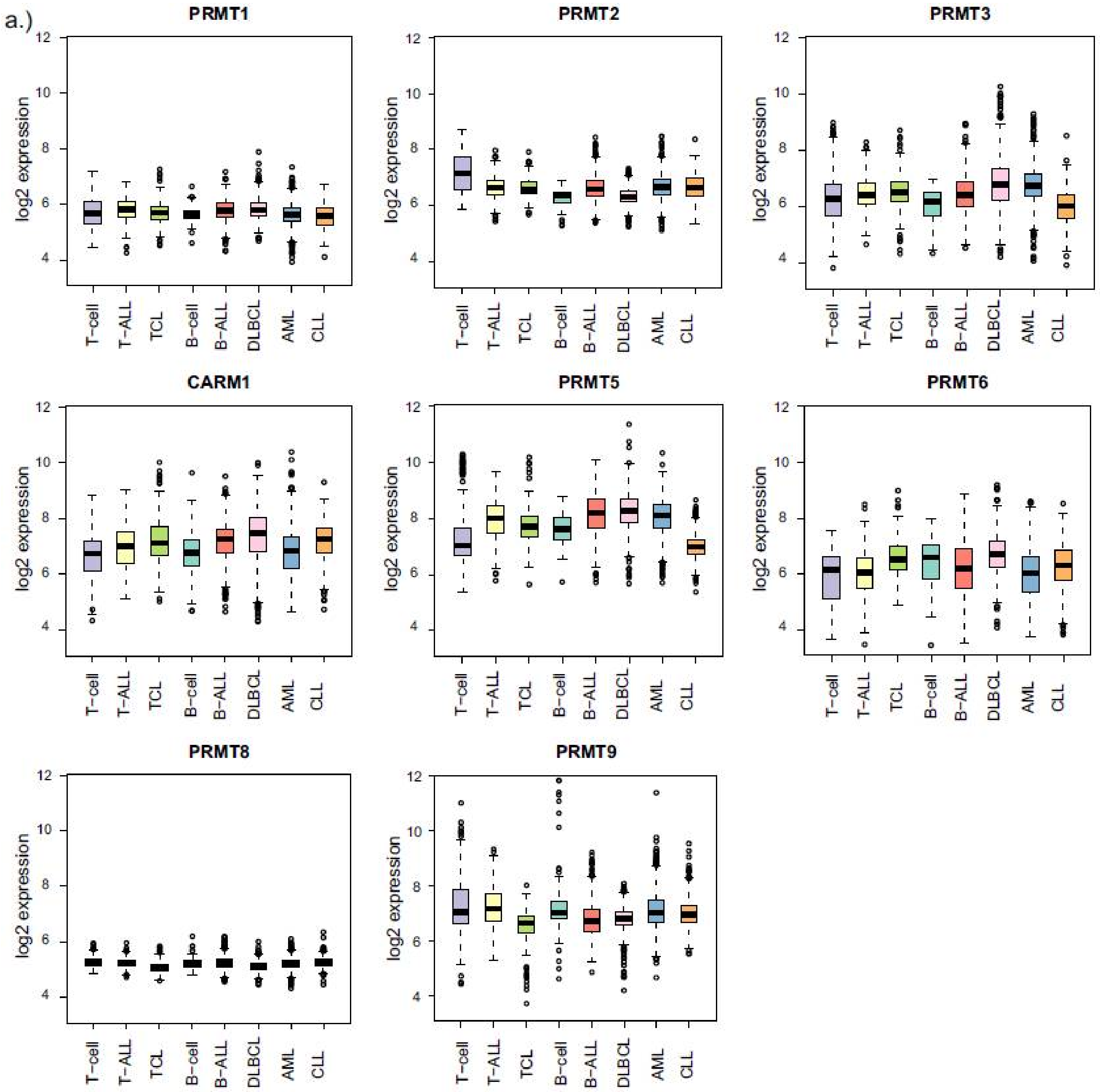

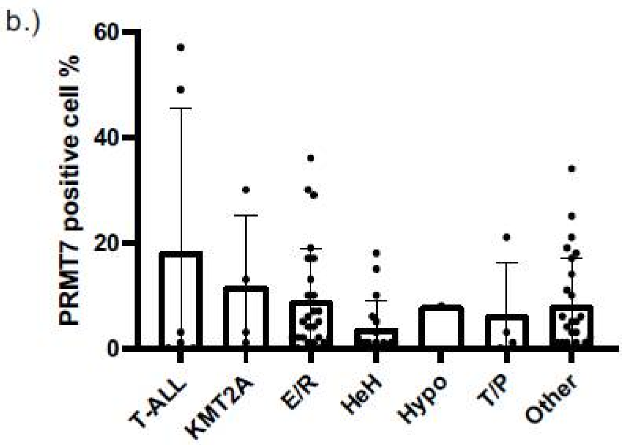

Figure S1. Expression of the *PRMT* family genes in (**a**) leukemias, lymphomas and healthy cells, and in. (**b**) expression of PRMT7 protein in trephine biopsy samples in subtypes of B-ALL and T-ALL (no statistically significant differences in expressions when compared PRMT7 expression in T-ALL cells to B-ALL subtypes).

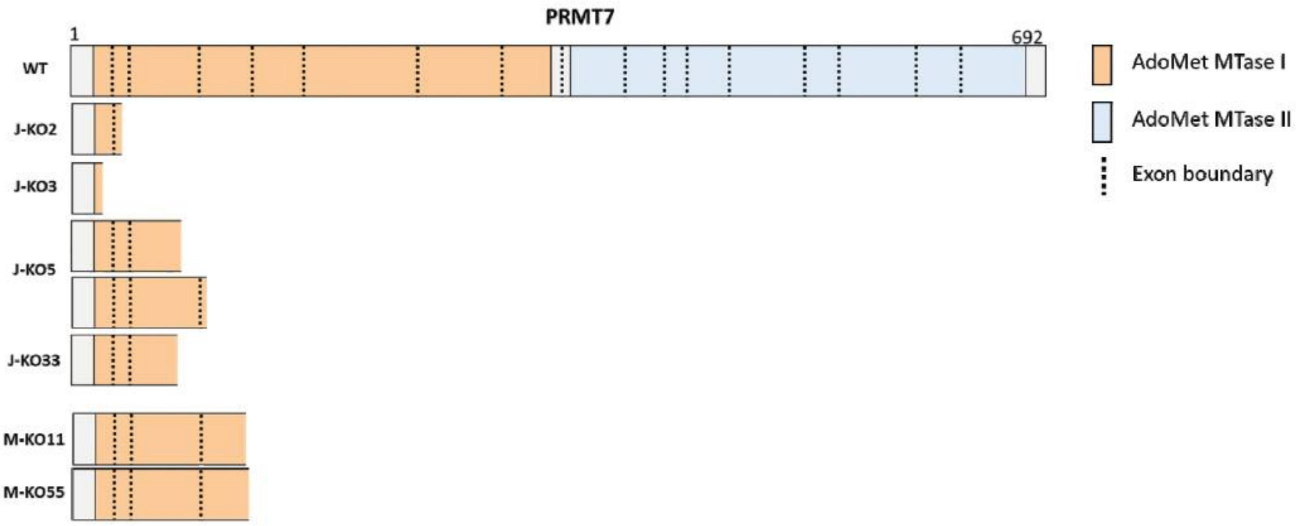

Cell line	Mutation	Amino acid changes	Position of STOP in WT/mut	Position (AA) of STOP in WT/mut	Prediction (Mutation taster)
J-KO2	chr16:68349960_68349960delC	H27Tfs20*	2079/138	692/46	Disease causing
J-KO3	chr16:68349960_68349969delCCACCAGGAG	Y26*	2079/78	692/26	Disease causing
J-KO5	chr16:68358662_68358663insCGGTC<br>chr16:68358661_68358662insA	I71Gfs*12<br>D70Efs*32	2079/246<br>2079/303	692/82<br>692/101	Disease causing
J-KO33	chr16:68358662_68358662delA<br>chr16:68358663_68358663delC	D70Afs*11<br>D70Efs*11	2079/240<br>2079/240	692/80<br>692/80	Disease causing
M-KO11	chr16:68363063_68363064insGA<br>chr16:68363064_68363064delG	T127Rfs*3<br>T127Lfs*2	2079/387<br>2079/284	692/129<br>692/123	Disease causing
M-KO55	chr16:68363066_68363067insAGCCCCAG<br>chr16:68363066_68363067delCT	V128Afs*4<br>T127Sfs*6	2079/393<br>2079/396	692/131<br>692/132	Disease causing

Figure S2. Schematic presentation of the consequences of CRISPR-Cas9 mediated genetic mutations of *PRMT7* in the Jurkat and Molt-4 cell lines.

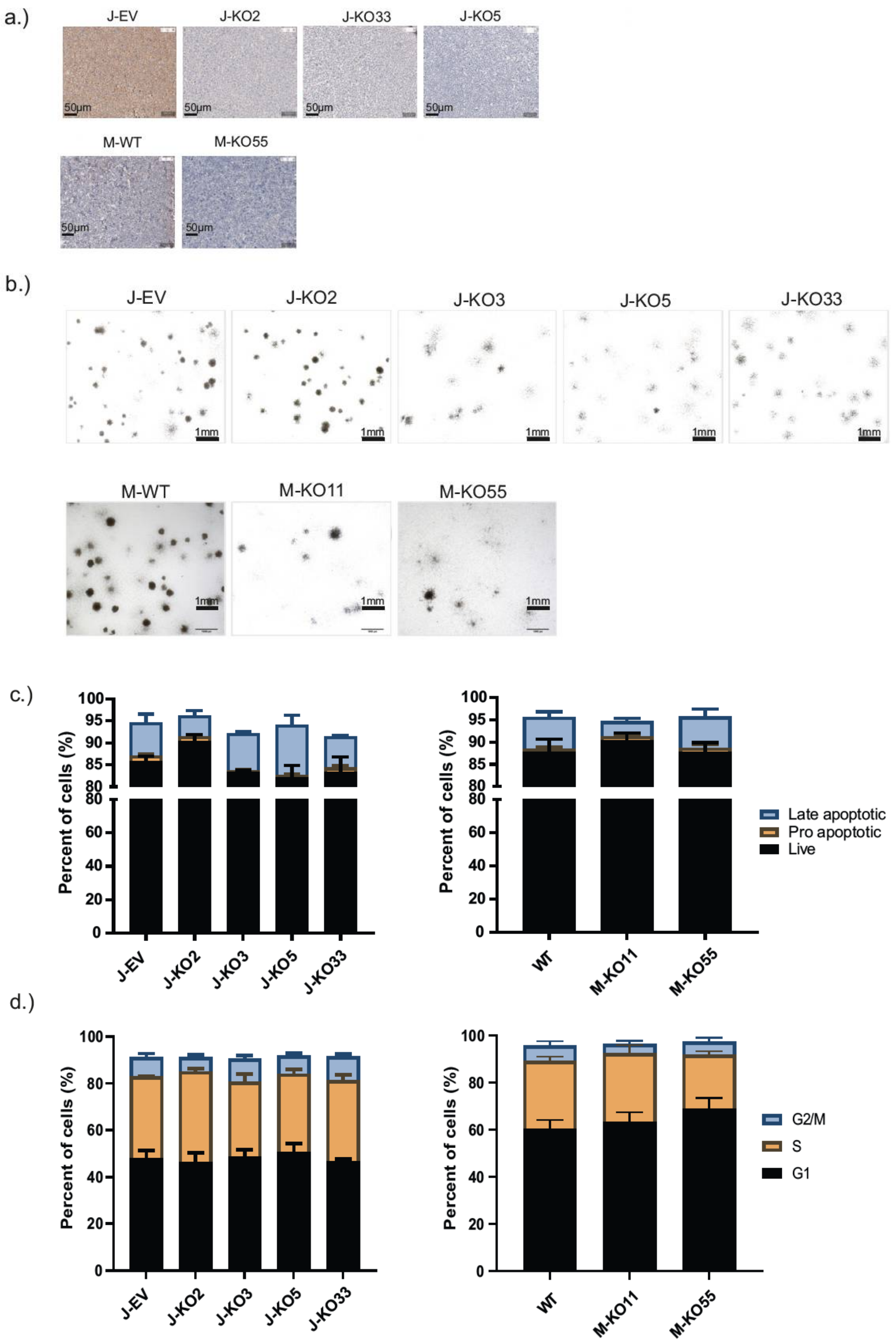

Figure S3. (**a**) Immunohistochemical staining (scale bar 50 µM) showing PRMT7 expression in J-EV, J-KO2, J-KO5, J-KO33, M-WT and M-KO55 cell lines. Brown color indicates positivty to the PRMT7, and blue marks PRMT7 negative cells. (**b**) Effects of *PRMT7* knockout on colony formation, (**c**) apoptosis, and (**d**) cell cycle. Bar plots indicate the mean value and SEM ($n = 3$). J stands for Jurkat and M for Molt4 cell line, EV for empty vector, WT for wild type and KO for knockout cell lines.

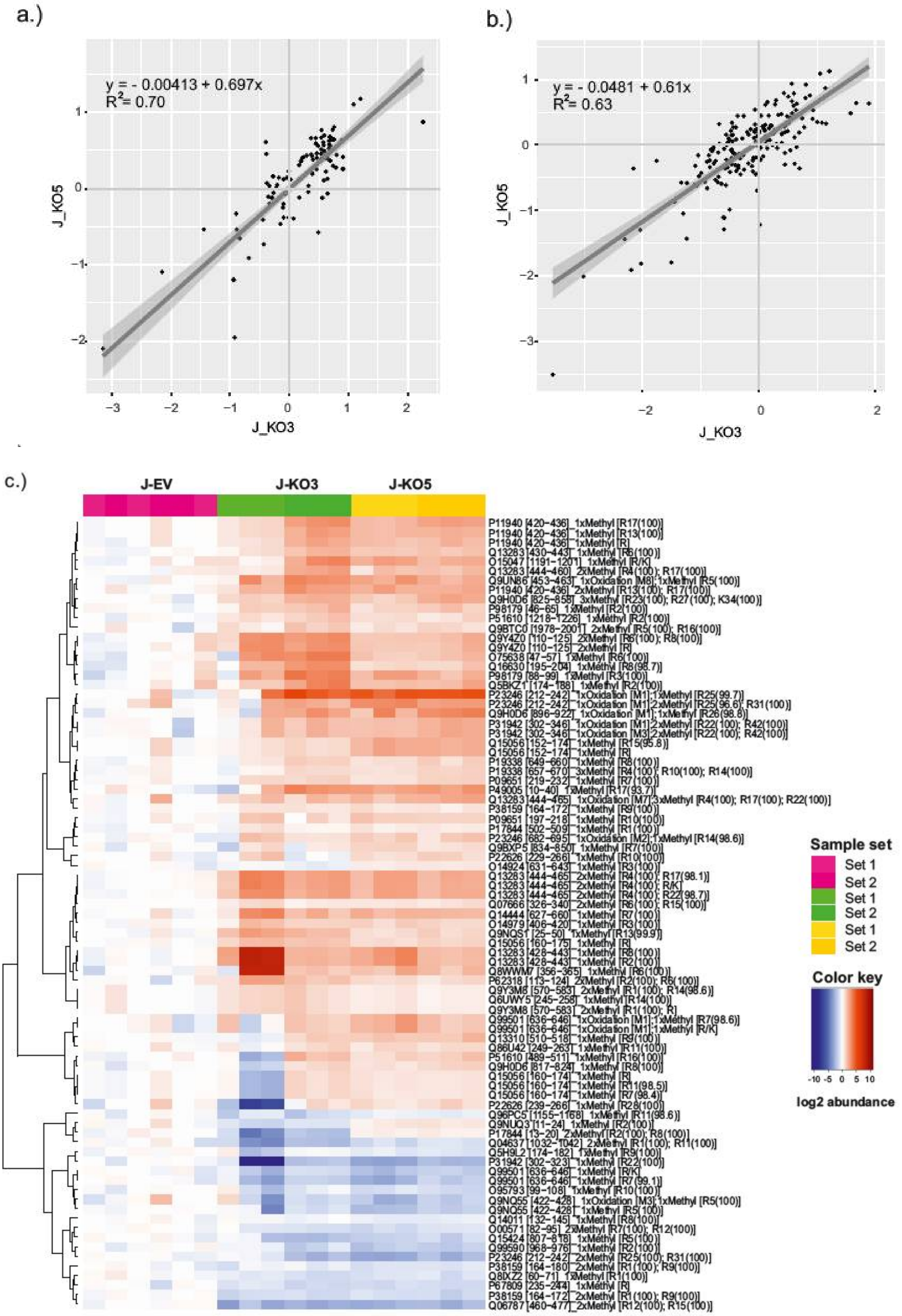

Figure S4. Correlation of arginine monomethylation findings in two PRMT7 knockout cell lines (J-KO3 and J-KO5) when using the (**a**) CORUM or (**b**) KEGG databases for the analysis. (**c**) Duplicate figure from Figure 4a, presenting row names for the heatmap.

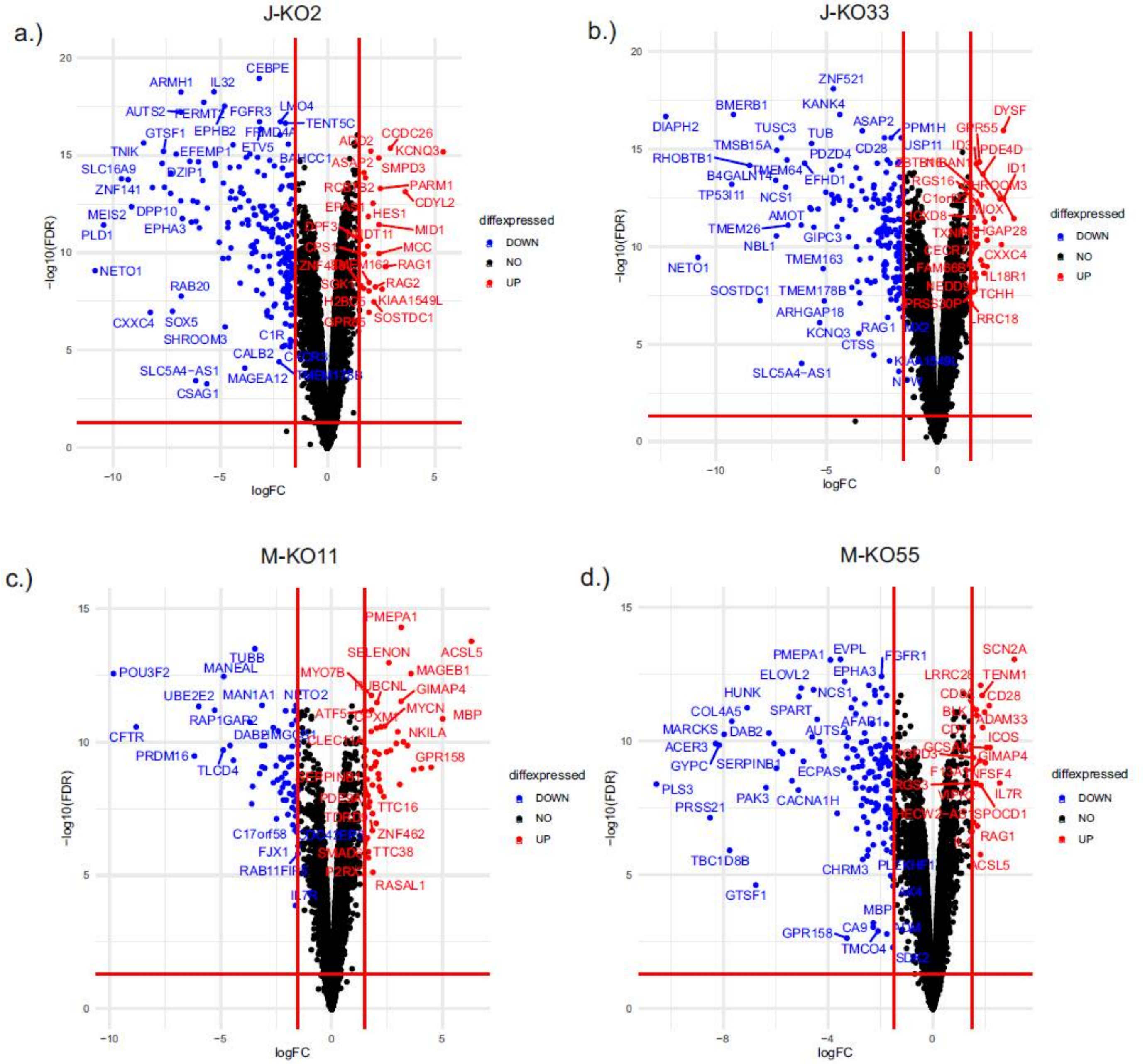

Figure S5. Effects of *PRMT7* knockout on gene expression of T-ALL cells. Volcano plots showing the differentially expressed genes (log2FC ≥ ±1.5, FDR < 0.05) in (**a**) J-KO2, (**b**) J-KO33, (**c**) M-KO11 and (**d**) M-KO55 cell lines. Red color indicates genes with significantly increased and blue with significantly decreased expression compared to unmodified cells. Three biological replicates were combined for the analysis for each cell line.

Figure 3b original blots.

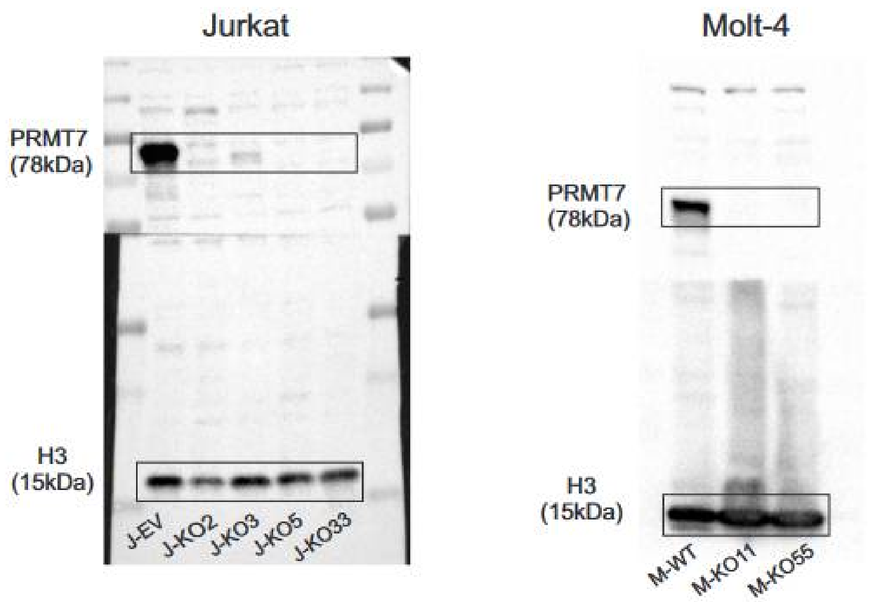

Figure S6. The uncropped Western blots.
